# Analytical modeling of trilayer graphene nanoribbon Schottky-barrier FET for high-speed switching applications

**DOI:** 10.1186/1556-276X-8-55

**Published:** 2013-01-30

**Authors:** Meisam Rahmani, Mohammad Taghi Ahmadi, Hediyeh Karimi Feiz Abadi, Mehdi Saeidmanesh, Elnaz Akbari, Razali Ismail

**Affiliations:** 1 Faculty of Electrical Engineering, Universiti Teknologi Malaysia (UTM), Johor Bahru, Johor 81310, Malaysia; 2 Electrical Engineering Department, Urmia University, Urmia 57135, Iran; 3 Malaysia-Japan International Institute of Technology (MJIIT), Universiti Teknologi Malaysia, Jalan Semarak, Kuala Lumpur 54100, Malaysia; 4 Centre for Artificial Intelligence and Robotics (CAIRO), Universiti Teknologi Malaysia, Jalan Semarak, Kuala Lumpur 54100, Malaysia

**Keywords:** Trilayer graphene nanoribbon (TGN), ABA and ABC stacking, TGN Schottky-barrier FET, High-speed switch

## Abstract

Recent development of trilayer graphene nanoribbon Schottky-barrier field-effect transistors (FETs) will be governed by transistor electrostatics and quantum effects that impose scaling limits like those of Si *metal-oxide-semiconductor field-effect transistor*s. The current–voltage characteristic of a Schottky-barrier FET has been studied as a function of physical parameters such as effective mass, graphene nanoribbon length, gate insulator thickness, and electrical parameters such as Schottky barrier height and applied bias voltage. In this paper, the scaling behaviors of a Schottky-barrier FET using trilayer graphene nanoribbon are studied and analytically modeled. A novel analytical method is also presented for describing a switch in a Schottky-contact double-gate trilayer graphene nanoribbon FET. In the proposed model, different stacking arrangements of trilayer graphene nanoribbon are assumed as metal and semiconductor contacts to form a Schottky transistor. Based on this assumption, an analytical model and numerical solution of the junction current–voltage are presented in which the applied bias voltage and channel length dependence characteristics are highlighted. The model is then compared with other types of transistors. The developed model can assist in comprehending experiments involving graphene nanoribbon Schottky-barrier FETs. It is demonstrated that the proposed structure exhibits negligible short-channel effects, an improved on-current, realistic threshold voltage, and opposite subthreshold slope and meets the International Technology Roadmap for Semiconductors near-term guidelines. Finally, the results showed that there is a fast transient between on-off states. In other words, the suggested model can be used as a high-speed switch where the value of subthreshold slope is small and thus leads to less power consumption.

## Background

Graphene, as a single layer of carbon atoms with hexagonal symmetry and different types such as monolayer, bilayer, trilayer, and multilayers, has attracted new research attention. Very high carrier mobility can be achieved from graphene-based materials which makes them a promising candidate for nanoelectronic devices [1,2]. Recently, electron and hole mobilities of a suspended graphene have reached as high as 2 × 10^5^ cm^2^/V·s [3]. Also, ballistic transport has been observed at room temperature in these materials [3]. Layers of graphene can be stacked differently depending on the horizontal shift of graphene planes [4,5]. Every individual multilayer graphene sequence behaves like a new material, and different stacking of graphene sheet lead to different electronic properties [3,6,7]. In addition, the configuration of graphene layers plays a significant role to realize either metallic or semiconducting electronic behavior [4,8,9].

**Figure 1:**
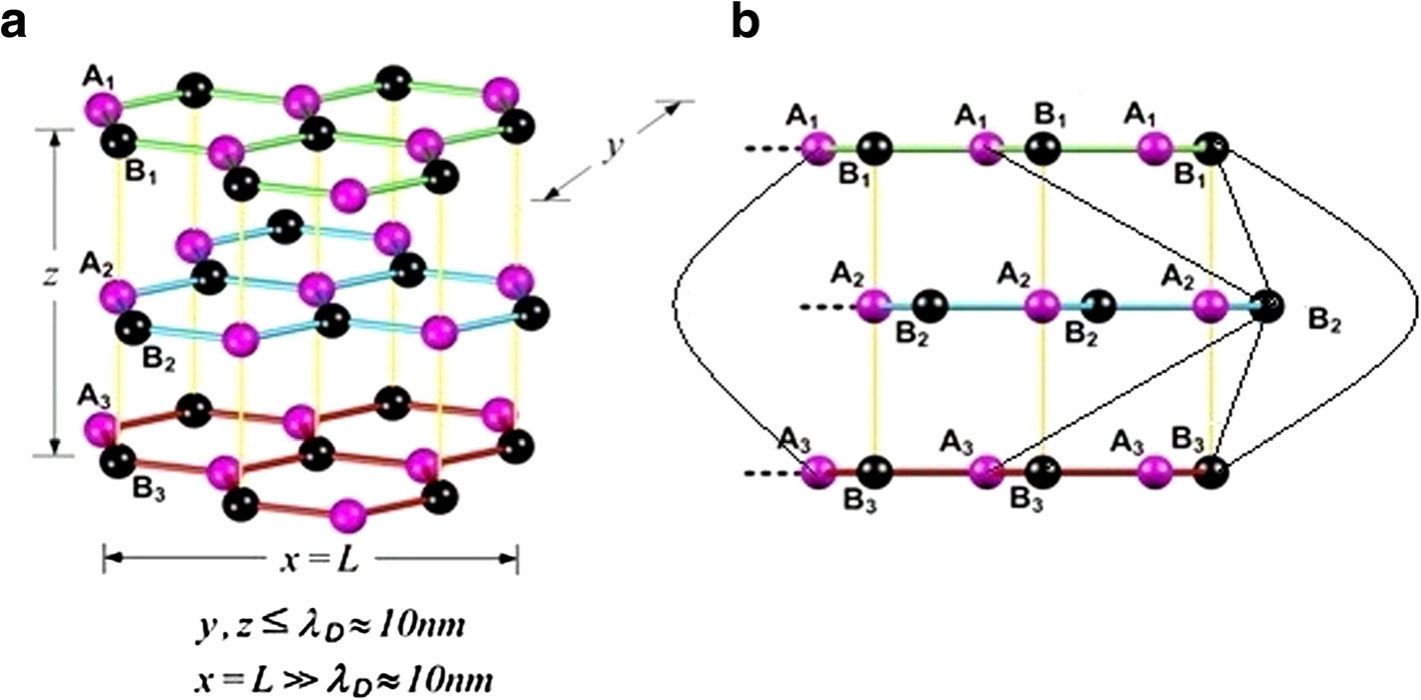
**TGN.** (**a**) As a one-dimensional material with quantum confinement effect on two Cartesian directions. (**b**) ABA-stacked [17].

Trilayer graphene nanoribbon (TGN), as a one-dimensional (1D) material, is the focus of this study. The quantum confinement effect will be assumed in two directions. In other words, only one Cartesian direction is greater than the de Broglie wavelength (10 nm). As shown in Figure 1a, because of the quantum confinement effect, a digital energy is taken in the *y* and *z* directions, while an analog type in the *x* direction. It is also remarkable that the electrical property of TGN is a strong function of interlayer stacking sequences [10]. Two well-known forms of TGN with different stacking manners are understood as ABA (Bernal) and ABC (rhombohedral) [11]. The simplest crystallographic structure is hexagonal or AA stacking, where each layer is placed directly on top of another; however, it is unstable. AB (Bernal) stacking is the distinct stacking structure for bilayers. For trilayers, it can be formed as either ABA, as shown in Figure 1, or ABC (rhombohedral) stacking [1,12]. Bernal stacking (ABA) is a common hexagonal structure which has been found in graphite. However, some parts of graphite can also have a rhombohedral structure (the ABC stacking) [6,13]. The band structure of ABA-stacked TGNs can be assumed as a hybrid of monolayer and bilayer graphene band structures. The perpendicular external applied electric or magnetic fields are expected to induce band crossing variation in Bernal-stacked TGNs [14-16]. Figure 1 indicates that the graphene plane being a two-dimensional (2D) honeycomb lattice is the origin of the stacking order in multilayer graphene with A and B and two non-equivalent sublattices.

As shown in Figure 1, a TGN with ABA stacking has been modeled in the form of three honeycomb lattices with pairs of equivalent sites as {A_1_,B_1_}, {A_2_,B_2_}, and {A_3_,B_3_} which are located in the top, center, and bottom layers, respectively [11]. An effective-mass model utilizing the Slonczewski-Weiss-McClure parameterization [17] has been adopted, where every parameter can be compared with a relevant parameter in the tight-binding model. The stacking order is related to the electronic low-energy structure of 3D graphite-based materials [18,19]. Interlayer coupling has been found to also affect the device performance, which can be decreased as a result of mismatching the A-B stacking of the graphene layers or rising the interlayer distance. A weaker interlayer coupling may lead to reduced energy spacing between the subbands and increased availability of more subbands for transfer in the low-energy array. Graphene nanoribbon (GNR) has been incorporated in different nanoscale devices such as interconnects, electromechanical switches, Schottky diodes, tunnel transistors, and field-effect transistors (FETs) [20-24]. The characteristics of the electron and hole energy spectra in graphene create unique features of graphene-based Schottky transistors. Recently, the fabrication and experimental studies as well as a hypothetical model of G-Schottky transistors have been presented [25]. The studies have focused towards the properties of TGN, and a tunable three-layer graphene single-electron transistor was experimentally realized [6,26].

**Figure 2:**
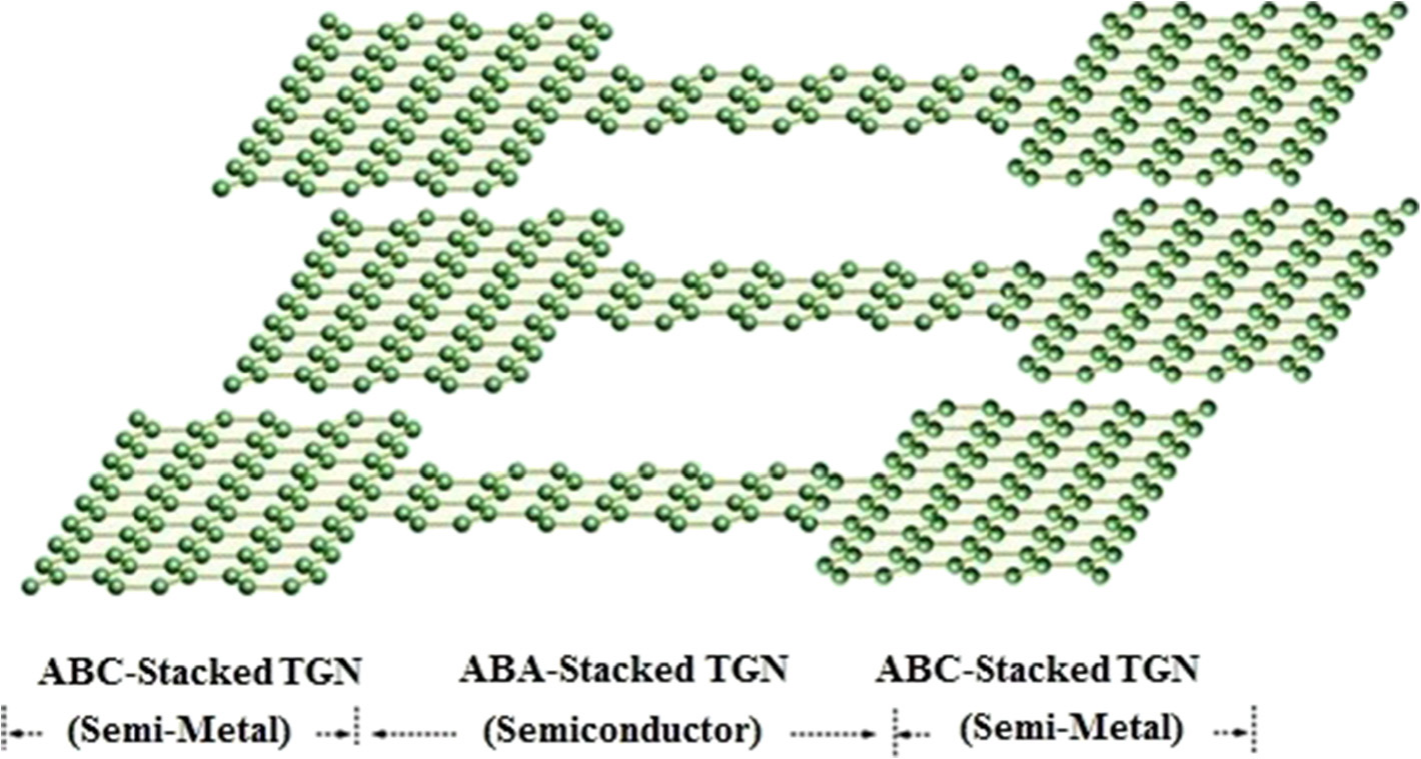
Schematic of TGN SB contacts.

In this paper, a model for TGN Schottky-barrier (SB) FET is analyzed which can be assumed as a 1D device with width and thickness less than the de Broglie wavelength. The presented analytical model involves a range of nanoribbons placed between a highly conducting substrate with the back gate and the top gate controlling the source-drain current. The Schottky barrier is defined as an electron or hole barrier which is caused by an electric dipole charge distribution related to the contact and difference created between a metal and semiconductor under an equilibrium condition. The barrier is found to be very abrupt at the top of the metal due to the charge being mostly on the surface [27-31]. TGN with different stacking sequences (ABA and ABC) indicates different electrical properties, which can be used in the SB structure. This means that by engineering the stack of TGN, Schottky contacts can be designed, as shown in Figure 2. Between two different arrangements of TGN, the semiconducting behavior of the ABA stacking structure has turned it into a useful and competent channel material to be used in Schottky transistors [32].

**Figure 3:**
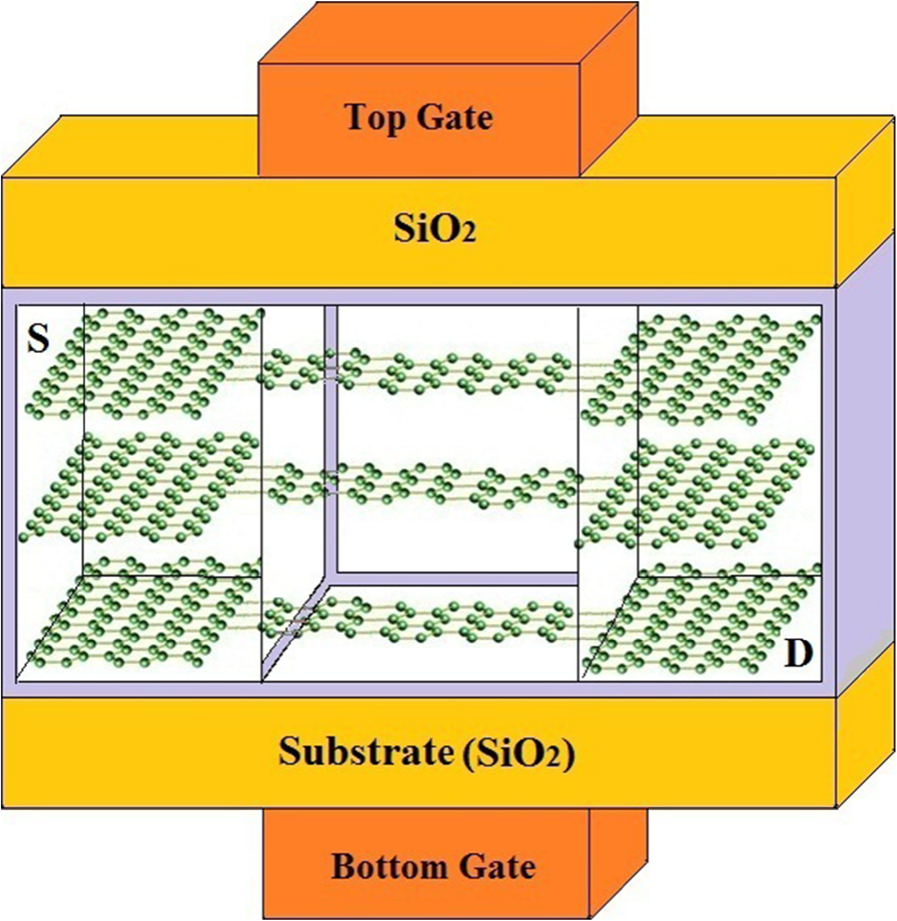
Schematic representation of TGN SB FET.

In fact, the TGN with ABC stacking shows a semimetallic behavior, while the ABA-stacked TGN shows a semiconducting property [32]. A schematic view of TGN SB FET is illustrated in Figure 3, in which ABA-stacked TGN forms the channel between the source and drain contacts. The contact size has a smaller effect on the double-gate (DG) GNR FET compared to the single-gate (SG) FET.

Due to the fact that the GNR channel is sandwiched or wrapped through by the gate, the field lines from the source and drain contacts were seen to be properly screened by the gate electrodes, and therefore, the source and drain contact geometry has a lower impact. The operation of TGN SB FET is followed by the creation of the lateral semimetal-semiconductor-semimetal junction under the controlling top gate and relevant energy barrier.

## Methods

### TGN SB FET model

**Figure 4:**
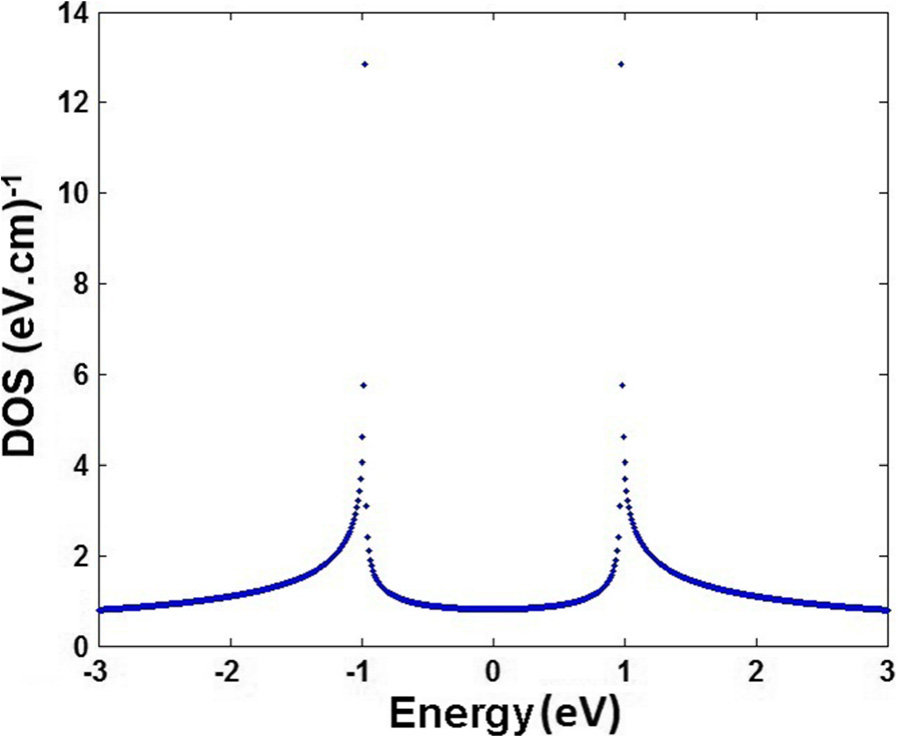
The DOS of the TGN with ABA stacking.

The scaling behaviors of TGN SB FET are studied by self-consistently solving the energy band structure equation in an atomistic basis set. In order to calculate the energy band structure of ABA-stacked TGN, the spectrum of full tight-binding Hamiltonian technique has been adopted [33-37]. The presence of electrostatic fields breaks the symmetry between the three layers. Using perturbation theory [38] in the limit of *υ*_*F*_|*k*| « *V* « *t*_⊥_ gives the electronic band structure of TGN as [35,39]

(1) $$E_{k}=\pm \left(\mathrm{\alpha k}-\beta k^{3}\right)\text{,}$$

where *k* is the wave vector in the *x* direction, $\alpha =\frac{v_{f}.V}{t_{⊥}\sqrt{2}},\beta =\frac{v_{f}{}^{3}}{t_{⊥}\sqrt{2}V}$, *t*_⊥_ is the hopping energy, *ν*_f_ is the Fermi velocity, and *V* is the applied voltage. The response of ABA-stacked TGN to an external electric field is different from that of mono- or bilayer graphene. Rather than opening a gap in bilayer graphene, this tuned the magnitude of overlap in TGN. Based on the energy dispersion of biased TGN, wave vector relation with the energy (*E*-*k* relation) shows overlap between the conduction and valence band structures, which can be controlled by a perpendicular external electric field [6,39]. The band overlap increases with increasing external electric field which is independent of the electric field polarity. Moreover, it is shown that the effective mass remains constant when the external electric field is increased [3,33]. As an essential parameter of TGNs, density of states (DOS) reveals the availability of energy states, which is defined as in [40,41]. To obtain this amount, derivation of energy over the wave vector is required. Since DOS shows the number of available states at each energy level which can be occupied, therefore, DOS, as a function of wave vector, can be modeled as [39]

(2) $$\mathrm{DOS}\left(E\right)=\frac{1}{A-\frac{B}{{\left(\mathrm{DE}+\sqrt{F+E^{2}}\right)}^{\frac{2}{3}}}-C{\left(\mathrm{DE}+\sqrt{F+E^{2}}\right)}^{\frac{2}{3}}}\text{,}$$

where *E* is the energy band structure and *A*, *B*, *C*, *D*, and *F* are defined as *A* = −6.2832*α*, *B* = 14.3849*α*^2^*β*, $C=\frac{2.7444}{\beta }$, *D* = −9*β*^2^, and $\;F=\frac{-0.1690\alpha^{3}}{\beta }$. As shown in Figure 4, the DOS for ABA-stacked TGN at room temperature is plotted. As illustrated, the low-DOS spectrum exposes two prominent peaks around the Fermi energy [39].

The electron concentration is calculated by integrating the Fermi probability distribution function over the energy as in [42]. Biased ABA-stacked TGN carrier concentration is modified as [43]

(3) $$n=\overset{\eta }{\underset{0}{\int }}\frac{\left(k_{B}T\right)\mathrm{dx}}{\left\{A-\frac{B}{{\left(k_{B}T\right)}^{\frac{2}{3}}{\left\{D\left(x+M\right)+\sqrt{N+{\left(x+M\right)}^{2}}\right\}}^{\frac{2}{3}}}-C{\left(k_{B}T\right)}^{\frac{2}{3}}{\left\{D\left(x+M\right)+\sqrt{N+{\left(x+M\right)}^{2}}\right\}}^{\frac{2}{3}}\right\}\left(1+\exp\left(x-\eta \right)\right)}\text{,}$$

where $x=\frac{E-E_{c}}{k_{B}T}$, the normalized Fermi energy is $\eta =\frac{-E_{c}+E_{f}}{k_{B}T}$, and *M* and *N* are defined as $M=\frac{E_{c}}{k_{B}T}$ and $\;N=\frac{F}{{\left(k_{B}T\right)}^{2}}$. Based on this model, ABA-stacked TGN carrier concentration is a function of normalized Fermi energy (*η*). The conductance of graphene at the Dirac point indicates minimum conductance at a charge neutrality point which depends on temperature. For a 1D TGN FET, the GNR channel is assumed to be ballistic. The current from source to drain can be given by the Boltzmann transport equation in which the Landauer formula has been adopted [44,45]. The number of modes in corporation with the Landauer formula indicates conductance of TGN that can be written as [32]

(4) $$\begin{array}{l} G=\frac{2\alpha q^{2}}{\mathrm{lh}}\overset{+\infty }{\underset{-\infty }{\int }}\left(-\frac{d}{\mathrm{dE}}\left(\frac{1}{1+e^{\frac{E-E_{F}}{K_{B}T}}}\right)\right)\mathrm{dE}\\ \;+\frac{-6\beta q^{2}}{\mathrm{lh}}\overset{+\infty }{\underset{-\infty }{\int }}k^{2}\left(-\frac{d}{\mathrm{dE}}\left(\frac{1}{1+e^{\frac{E-E_{F}}{K_{B}T}}}\right)\right)\mathrm{dE}\text{,} \end{array}$$

where the momentum (*k*) can be derived by using Cardano's solution for cubic equations [46]. Equation 4 can be assumed in the form *G = N*_1_*G*_1_*+ N*_2_*G*_2_, where *N*_1_ = 2*αq*^2^/*lh* and *N*_2_ = −6*βq*^2^/*lh*. Since *G*_1_ is an odd function, its value is equivalent to zero. Therefore, *G* = *N*_2_*G*_2_[32], where

(5) $$\begin{array}{l} G_{2}=\overset{+\infty }{\underset{-\infty }{\int }}\left({\left(-\frac{E}{2\beta }+\sqrt{{\left(\frac{-\alpha }{3\beta }\right)}^{3}+{\left(\frac{E}{2\beta }\right)}^{2}}\right)}^{\frac{1}{3}}\right)\\ {\left(\;,+,{\left(-\frac{E}{2\beta }-\sqrt{{\left(\frac{-\alpha }{3\beta }\right)}^{3}+{\left(\frac{E}{2\beta }\right)}^{2}}\right)}^{\frac{1}{3}}\right)}^{2}\\ \;x\left(-\frac{d}{\mathrm{dE}}\left(\frac{1}{1+e^{\frac{E-E_{F}}{k_{B}T}}}\right)\right)\mathrm{dE}\text{.} \end{array}$$

This equation can be numerically solved by employing the partial integration method and using the simplification form, where *x* = (*E* − Δ)/*k*_B_*T* and *η* = (*E*_F_ − Δ)/*k*_B_*T*. Thus, the general conductance model of TGN will be obtained [32] as

(6) $$G_{2}=-\overset{+\infty }{\underset{-\infty }{\int }}\left\{\begin{array}{c} \left(\frac{2k_{B}T}{1+e^{x-\eta }}\right)\times \left(\frac{-\frac{k_{B}T}{2\beta }-\frac{xk_{B}T+\Delta }{4\beta^{2}\sqrt{-\frac{\alpha^{3}}{27\beta^{3}}+\frac{E^{2}}{4\beta^{2}}}}}{3{\left(-\frac{xk_{B}T+\Delta }{2\beta }-\sqrt{-\frac{\alpha^{3}}{27\beta^{3}}+\frac{{\left(xk_{B}T+\Delta \right)}^{2}}{4\beta^{2}}}\right)}^{2/3}}+\frac{-\frac{k_{B}T}{2\beta }+\frac{xk_{B}T+\Delta }{4\beta^{2}\sqrt{-\frac{\alpha^{3}}{27\beta^{3}}+\frac{E^{2}}{4\beta^{2}}}}}{3{\left(-\frac{xk_{B}T+\Delta }{2\beta }+\sqrt{-\frac{\alpha^{3}}{27\beta^{3}}+\frac{{\left(xk_{B}T+\Delta \right)}^{2}}{4\beta^{2}}}\right)}^{2/3}}\right)\\ \times \left({\left(-\frac{xk_{B}T+\Delta }{2\beta }-\sqrt{-\frac{\alpha^{3}}{27\beta^{3}}+\frac{{\left(xk_{B}T+\Delta \right)}^{2}}{4\beta^{2}}}\right)}^{1/3}+{\left(-\frac{xk_{B}T+\Delta }{2\beta }+\sqrt{-\frac{\alpha^{3}}{27\beta^{3}}+\frac{{\left(xk_{B}T+\Delta \right)}^{2}}{4\beta^{2}}}\right)}^{1/3}\right) \end{array}\right\}\mathrm{dx}\text{.}$$

It can be seen that the conductivity of TGN increases by raising the magnitude of gate voltage. In the Schottky contact, electrons can be injected directly from the metal into the empty space in the semiconductor. When electrons flow from the valence band of the semiconductor into the metal, there would be a result similar to that for holes injected into the semiconductor. So, the establishment of an excess minority carrier hole in the vicinity is observed [28]. The current moves mainly from the drain to the source which consists of both drift and diffusion currents. The created 2D anticipated framework is expected to cause an explicit analytical current equation in the subthreshold system. Considering the weak inversion region, the diffusion current is mainly dominated and relative to the electron absorption at the virtual cathode [47]. A GNR FET is a voltage-controlled tunnel barrier device for both the Schottky and doped contacts.

The drain current through the barrier consists of thermal and tunneling components [48]. The effect of quantum tunneling and electrostatic short channel is not treated, which makes it difficult to study scaling behaviors and ultimate scaling limits of GNR SB FET where the tunneling effect cannot be ignored [20]. The tunneling current is the main component of the whole current which requires the use of the quantum transport. Close to the source within the band gap, carriers are injected into the channel from the source [49]. In fact, the tunneling current plays a very important role in a Schottky contact device.

The proposed model includes tunneling current through the SB at the contact interfaces, appropriately capturing the impact of arbitrary electrical and physical factors. The behavior of the proposed transistor over the threshold region is obtained by modulating the tunneling current through the SBs at the two ends of the channel [20]. The effect of charges close to the source for a SB FET is more severe because they have a significant effect on the SB and the tunneling possibility. When the charge impurity is situated at the center of the channel of a SB FET, the electrons are trapped by the positive charge and the source-drain current is decreased. If the charges are situated close to the drain, the electrons will collect near the drain. In this situation, low charge density near the source decreases the potential barrier at the beginning of the channel, which opens up the energy gap more for the flow of electrons from the source to the channel [50].

Electrons moving from the metal into the semiconductor can be defined by the electron current density *J*_m→s_, whereas the electron current density *J*_s→m_ refers to the movement of electrons from the semiconductor into the metal. What determines the direction of electron flow depends on the subscripts of the current. In other words, the conventional current direction is opposite to the electron flow. *J*_s→m_ is related to the concentration of electrons with velocity in the *x* direction to subdue the barrier [28]:

(7) $$J_{s\to m}=e\overset{+\infty }{\underset{-\infty }{\int }}\nu_{x}\mathrm{dn}\text{,}$$

where *e* is the magnitude of the electronic charge and *ν*_x_ is the carrier velocity in the direction of transport:

(8) $$J_{\mathrm{total}}=J_{m\to s}-\frac{dJ_{s\to m}}{\mathrm{dx}}\text{.}$$

High carrier mobility reported from experiments on graphene leads to assume a complete ballistic carrier transport in the TGN, which means that the average probability of injected electron at one end that will transmit to the other end is approximately equal to 1:

(9) $$J_{\mathrm{total}}=J_{m\to s}=J_{s\to m}\text{.}$$

Kinetic energy, as a main parameter, is considered over the Fermi level, and the current density-voltage response of the TGN SB FET device is determined with respect to the carrier density and its kinetic energy as

(10) $$J_{s\to m}=\frac{\sqrt{2}e}{\sqrt{m^{*}}}\overset{+\infty }{\underset{-\infty }{\int }}\frac{{\left(k_{B}T\right)}^{\frac{3}{2}}x^{\frac{1}{2}}\mathrm{dx}}{\left\{A-\frac{B}{{\left(k_{B}T\right)}^{\frac{2}{3}}{\left\{D\left(x+M\right)+\sqrt{N+{\left(x+M\right)}^{2}}\right\}}^{\frac{2}{3}}}-C{\left(k_{B}T\right)}^{\frac{2}{3}}{\left\{D\left(x+M\right)+\sqrt{N+{\left(x+M\right)}^{2}}\right\}}^{\frac{2}{3}}\right\}\left(1+\exp\left(x-\eta \right)\right)}\text{,}$$

where $\eta \approx \frac{V_{A}-V_{T}}{k_{B}T/e}$ (*V*_A_ is the applied bias voltage and *V*_T_ is the thermal voltage) [51]. The dependence of the drain current on the drain-source voltage is associated with the dependence of *η* on this voltage given by

(11) $$\eta =\overset{V_{\mathrm{DS}}}{\underset{0}{\int }}\frac{\left(V_{\mathrm{GT}}-V\left(y\right)\right)e}{K_{B}T}\mathrm{dv}\text{,}$$

where *V*_GT_ = *V*_GS_ − *V*_T_ and *V*(y) is the voltage of channel in the *y* direction. By solving Equation 11, the normalized Fermi energy can be defined as

(12) $$\eta =\frac{e}{K_{B}T}\left[V_{\mathrm{GT}}V_{\mathrm{DS}}-\frac{V_{\text{DS}}^{2}}{2}\right]\text{.}$$

In order to obtain an analytical relation for the contact current, an explicit analytical equation for the electric potential distribution along the TGN is presented. The channel current is analytically derived as a function of various physical and electrical parameters of the device including effective mass, length, temperature, and applied bias voltage. According to the relationship between a current and its density, the current–voltage response of a TGN SB FET, as a main characteristic, is modeled as

(13) $$I=\frac{\sqrt{2}\mathrm{el}}{\sqrt{m^{*}}}\overset{+\infty }{\underset{-\infty }{\int }}\frac{{\left(k_{B}T\right)}^{\frac{3}{2}}x^{\frac{1}{2}}\mathrm{dx}}{\left\{A-\frac{B}{{\left(k_{B}T\right)}^{\frac{2}{3}}{\left\{D\left(x+M\right)+\sqrt{N+{\left(x+M\right)}^{2}}\right\}}^{\frac{2}{3}}}-C{\left(k_{B}T\right)}^{\frac{2}{3}}{\left\{D\left(x+M\right)+\sqrt{N+{\left(x+M\right)}^{2}}\right\}}^{\frac{2}{3}}\right\}\left(1+\exp\left(x-\frac{e}{K_{B}T}\left[V_{\mathrm{GT}}V_{\mathrm{DS}}-\frac{V_{\mathrm{DS}}{}^{2}}{2}\right]\right)\right)}$$

where *l* is the length of the channel.

## Results and discussion

**Figure 5:**
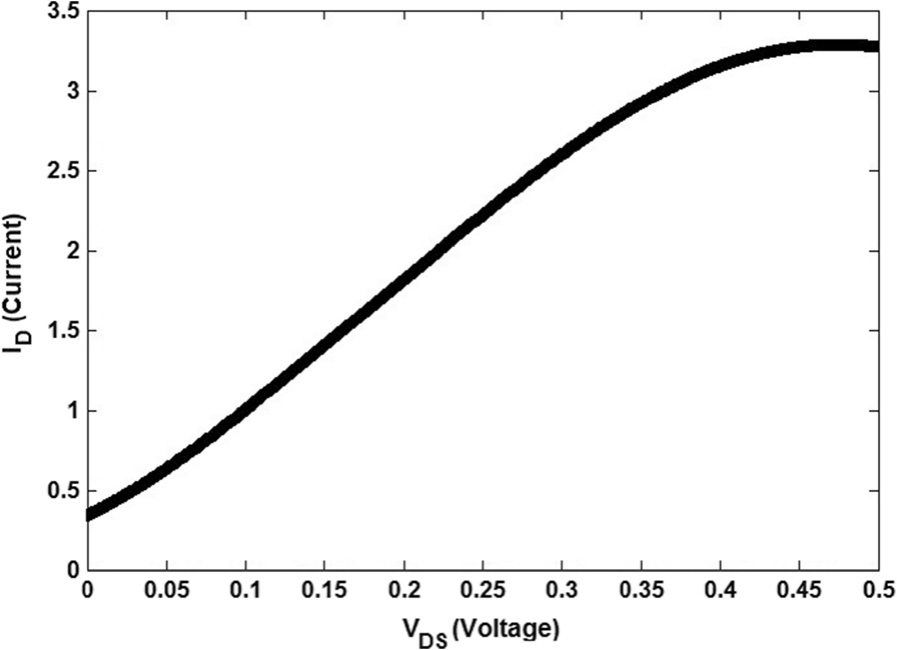
**Simulated *I***_**D **_**(μA) versus *V***_**DS **_**(V) plots of TGN Schottky-barrier FET (*L *= 25 nm, *V***_**GS **_**= 0.5 V).**

In this section, the performance of the Schottky-contact double-gate TGN FET is studied. A novel analytical method is introduced to achieve a better understanding of the TGN SB switch devices. The results will be applied to identify how various device geometries provide different degrees of controlling transient between on-off states. The numerical solution of the presented analytical model in the preceding section was employed, and rectification current–voltage characteristic of TGN SB FET is plotted as shown in Figure 5.

**Figure 6:**
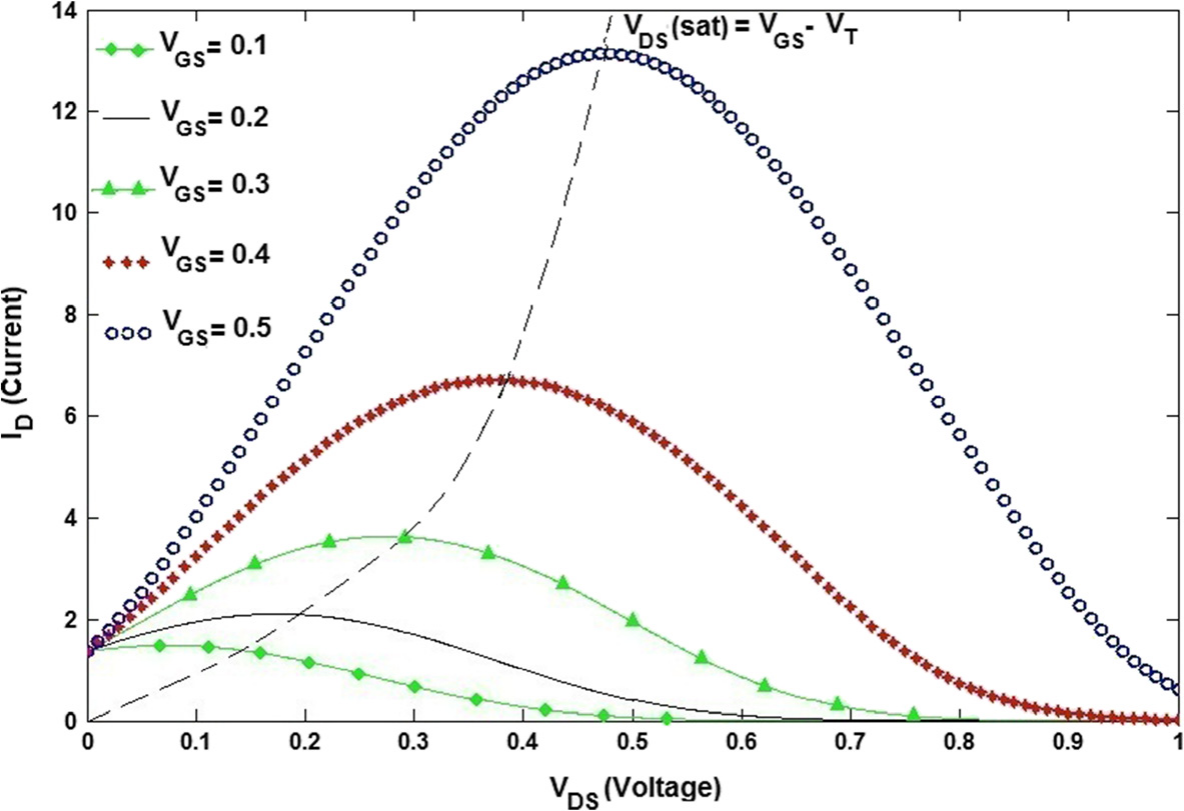
***I***_**D **_**(μA)-*V***_**DS **_**(V) characteristic of TGN SB FET at different values of *V***_**GS **_**for *L *= 100 nm.**

It further revealed that the engineering of SB height does not alter the qualitative ambipolar feature of the current–voltage characteristic whenever the gate oxide is thin. The reason is that the gate electrode could perfectly screen the field from the drain and source for a thin gate oxide (less than 10 nm). The SB whose thickness is almost the same as the gate insulator diameter is almost transparent. However, the ambipolar current–voltage (*I*-*V*) characteristic cannot be concealed by engineering the SB height when the gate insulator is thin. Lowering the gate insulator thickness and the contact size leads to thinner SBs and also greater on-current. Since the SB height is half of the band gap, the minimum currents exist at the gate voltage of *V*_G,min_ = 1/2*V*_D_, at which the conduction band that bends at the source extreme of the channel is symmetric to the valence band and also bends at the drain end of the channel, while the electron current is the same as the hole current. The consequence of attaining the least leakage current is the same as TGN SB FET with middle-gap SBs [23]. Raising the drain voltage leads to an exponential increase of the minimal leakage current which shows the importance of proper designing of the power supply voltage to ensure small leakage current. As depicted in Figure 6, the proposed model points out strong gate-source voltage dependence of the current–voltage characteristic showing that the *V*_GS_ increment effect will influence the drain current. In other words, as *V*_GS_ increases, a greater value of *I*_D_ results. As the drain voltage rises, the voltage drop through the oxide close to the drain terminal reduces, and this shows that the induced inversion charge density close to the drain also decreases [28]. The slope of the *I*_D_ versus *V*_DS_ curve will reduce as a result of the decrease in the incremental conductance of the channel at the drain. This impact is indicated in the *I*_D_-*V*_DS_ curve in Figure 6. If *V*_DS_ increases to the point that the potential drop across the oxide at the drain terminal is equal to *V*_T_, the induced inversion charge density is zero at the drain terminal. At that point, the incremental conductance at the drain is *nil*, meaning that the slope of the *I*_D_-*V*_DS_ curve is zero. We can write

(14) $$V_{\mathrm{DS}}\left(\mathrm{sat}\right)=V_{\mathrm{GS}}-V_{T}\text{,}$$

where *V*_DS_ (sat) is the drain-to-source voltage which is creating zero inversion charge density at the drain terminal. When *V*_DS_ is more than the *V*_DS_ (sat) value, the point in the channel where the inversion charge is zero moves closer to the source terminal [28]. In this case, electrons move into the channel at the source and pass through the channel towards the drain, and then at that point when the charge goes to zero, the electrons are infused into the space charge region where they are swept by the E-field to the drain contact. Compared to the original length *L*, the change in channel length Δ*L* is small, then the drain current will be regular for *V*_DS_ >*V*_DS_ (sat). The region of the *I*_D_-*V*_DS_ characteristic is referred to as the saturation region. When *V*_GS_ is changed, the *I*_D_-*V*_DS_ curve will also be changed. It was found that if *V*_GS_ increases, the initial slope of *I*_D_-*V*_DS_ will be raised. We can also infer from Equation 14 that the value of *V*_DS_ (sat) is a function of *V*_GS_. A family of curves is created for this n-channel enhancement-mode TGN SB FET, as shown in Figure 6.

**Figure 7:**
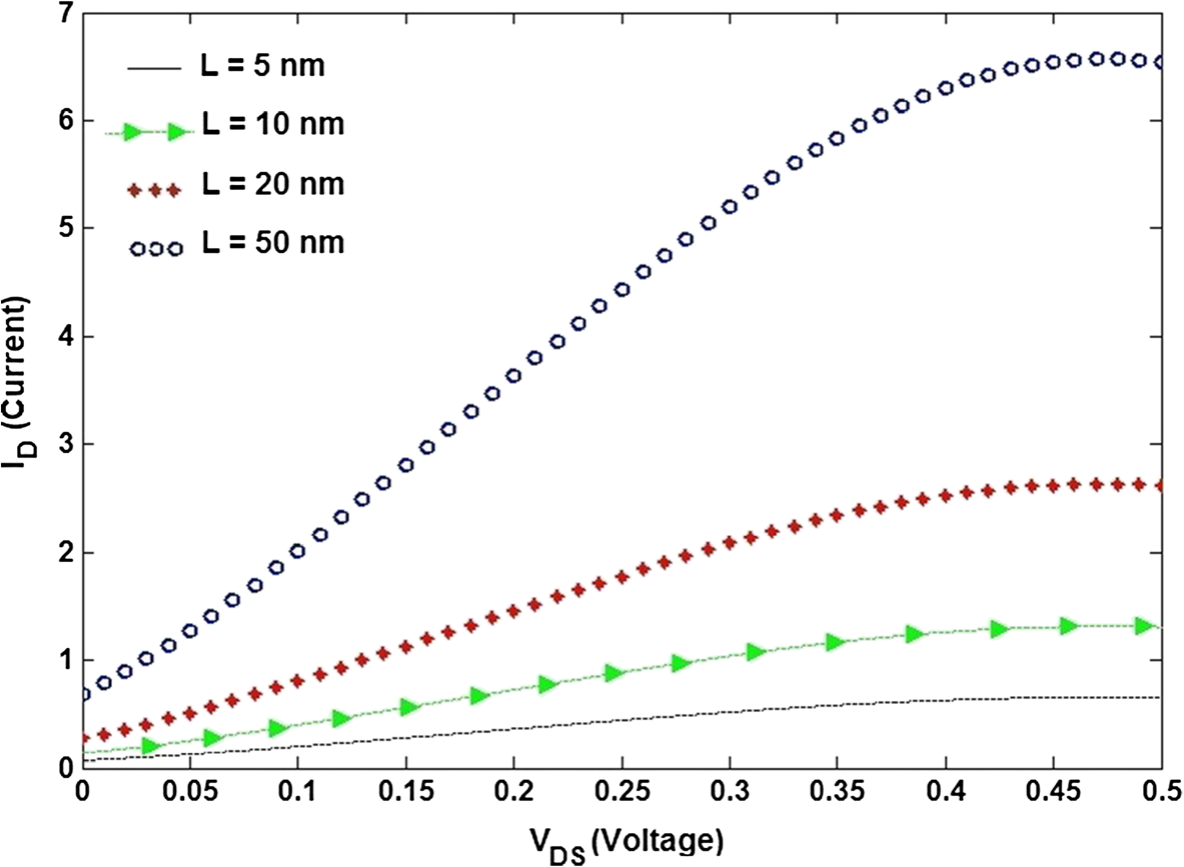
**Impact of the channel length scaling on the transfer characteristic for *V***_**GS **_**= 0.5 V.**

Also, it can be seen that by increasing *V*_GS_, the saturation current increases, showing the fact that a larger voltage drop occurs between the gate and the source contact. Also, there is a bigger energy value for carrier injection from the source contact channel [20]. The impact of power supply up-scaling decreases the SB length at the drain side, allowing it to be more transparent and resulting in more turn-on current to flow. Therefore, an acceptable performance comparable to the conventional behavior of a Schottky transistor is obtained. The scaling of the channel length improves gate electrostatic control, creating larger transconductance and smaller subthreshold swings. The effect of the channel length scaling on the *I*-*V* characteristic of TGN SB FET is investigated in Figure 7. It shows a similar trend when the gate-source voltage is changed. It can be seen that the drain current rises substantially as the length of the channel is increased from 5 to 50 nm.

To get a greater insight into the effect of increasing channel length on the increment of the drain current, two important factors, which are the transparency of SB and the extension of the energy window for carrier concentration, play a significant role [49,50]. For the first parameter, as the SB height and tunneling current are affected significantly by the charges close to the source of SB FET, the channel length effect on the drain current through the SB contact is taken into account in our proposed model. Moreover, when the center of the channel of the SB FET is unoccupied with the charge impurities, the drain-source current increases because of the fact that free electrons are not affected by positive charges [49]. The effect of the latter parameter appears at the beginning of the channel where the barrier potential decreases as a result of low charge density near the source. This phenomenon leads to widening the energy window and ease of electron flow from the source to the channel [50]. Furthermore, due to the long mean free path of GNR [52-55], the scattering effect is not dominant; therefore, increasing the channel length will result in a larger drain current.

For a channel length of 5 nm, direct tunneling from the source to drain results in a larger leakage current, and the gate voltage may rarely adjust the current. The transistor is too permeable to have a considerable disparity among on-off states. For a channel length of 10 nm, the drain current has improved to about 1.3 mA. The rise in the drain current is found to be more significant for channel lengths higher than 20 nm. That is, by increasing the channel length, there is a dramatic rise in the initial slope of *I*_D_ versus *V*_DS_. Also, based on the subthreshold slope model and the following simulated results, a faster device with opposite subthreshold slope or high on/off current ratio is expected. In other words, it can be concluded that there is a fast transient between on-off states. Increasing the channel length to 50 nm resulted in the drain current to increase by about 6.6 mA. The operation of the state-of-the-art short-channel TGN SB FET is found to be near the ballistic limit. Increasing further the channel length hardly changes neither the on-current or off-current nor the on/off current ratio. However, for a conventional *metal-oxide-semiconductor field-effect transistor (*MOSFET), raising the channel length may result in the channel resistance to proportionally increase. Therefore, in this case, down-scaling the channel length will result in significant loss of the on/off current ratio as compared to the SG device.

**Figure 8:**
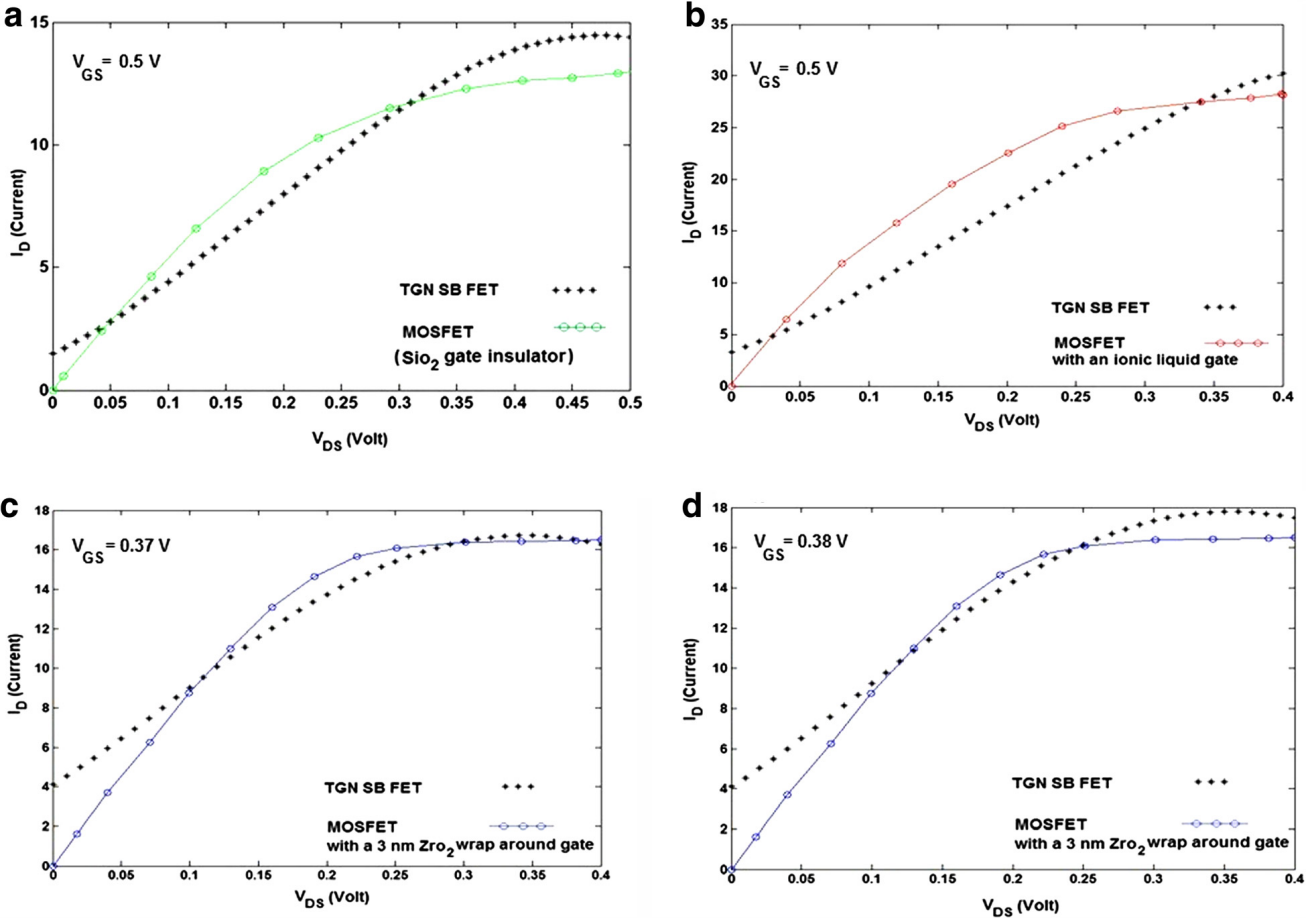
**Comparison between proposed model and typical *I*-*V *characteristics of other types of transistors.** (**a**) MOSFET with SiO_2_ gate insulator [50] (*V*_GS_ = 0.5V), (**b**) TGN MOSFET with an ionic liquid gate, *C*_ins_ >> *C*_q_[49] (*V*_GS_ = 0.5 V), (**c**) TGN MOSFET with a 3-nm ZrO_2_ wrap around gate, *C*_ins_*~ C*_q_[49] (*V*_GS_ = 0.37 V), (**d**) TGN MOSFET with a 3-nm ZrO_2_ wrap around gate, *C*_ins_ ~ *C*_q_[49] (*V*_GS_ = 0.38 V).

Figure 8 shows a comparative study of the presented model and the typical *I*-*V* characteristics of other types of transistors [49,50]. As depicted in Figure 8, the proposed model has a larger drain current than those transistors for some value of the drain-source voltages. The resultant characteristics of the presented model shown in Figure 8 are in close agreement with published results [49,50]. In Figure 8, DG geometry is assumed for the simulations instead of the SG geometry type.

In order to have a deep quantitative understanding of experiments involving GNR FETs, the proposed model is intended to aid in the design of such devices. The SiO_2_ gate insulator is 1.5 nm thick with a relative dielectric constant *K* = 3.9 [50] (Figure 8a). Furthermore, the gate-to-channel capacitance *C*_g_ is a serial arrangement of insulator capacitance *C*_ins_ and quantum capacitance *C*_q_ (equivalent to the semiconductor capacitance in conventional MOSFETs). Figure 8b shows a comparative study of the presented model and the typical *I*-*V* characteristic of a TGN MOSFET with an ionic liquid gate. The availability of the ionic liquid gating [49] that can be modeled as a wrap-around gate of a corresponding oxide thickness of 1 nm and a dielectric constant *ε*_r_ = 80 results in *C*_ins_ >>*C*_q_, and MOSFETs function close to the quantum capacitance limit, i.e., *C*_g_ ≈ *C*_q_[49]. As depicted in Figure 8c,d, the comparison study of the proposed model with a TGN MOSFET with a 3-nm ZrO_2_ wrap-around gate for two different values of *V*_GS_ is notable. A 3-nm ZrO_2_ (*ε*_r_ = 25) wrap-around gate has *C*_ins_ comparable to *C*_q_ for solid-state high-*κ* gating, and this is an intermediate regime among the MOSFET limit and *C*_q_ limit.

Recently, a performance comparison between the GNR SB FETs and the MOSFET-like-doped source-drain contacts has been carried out using self-consistent atomistic simulations [20,21,48-50,56,57]. The MOSFET demonstrates improved performance in terms of bigger on-current, larger on/off current ratio, larger cutoff frequency, smaller intrinsic delay, and better saturation behavior [21,50]. Disorders such as edge roughness, lattice vacancies, and ionized impurities have an important effect on device performance and unpredictability. This is because the sensitivity to channel atomistic structure and electrostatic environment is strong [50]. However, the intrinsic switching speed of the GNR SB FET is several times faster than that of the Si MOSFETs. This could lead to promising high-speed electronics applications, where the large leakage of the GNR SB FET is of fewer concerns [20]. An efficient functionality of the transistor with a doped nanoribbon has been noticed in terms of on/off current ratio, intrinsic switching delay, and intrinsic cutoff frequency [48].

Based on the presented model, comparable with the other experimental and analytical models, the on-state current of the MOSFET-like GNR FET is 1 order of magnitude higher than that of the TGN SB FET. This is because the gate voltage ahead of the source-channel flat band condition modulates both the thermal and tunnel components in the on-state of MOSFET-like GNR FET, while it modulates the tunnel barrier only of the metal Schottky-contact TGN FET that limits the on-state current. Furthermore, TGN SB FET device performance can be affected by interlayer coupling, which can be decreased by raising the interlayer distance or mismatching the A-B stacking of the graphene layers.

It is also noteworthy that MOSFETs operate in the region of subthreshold (weak inversion) as the magnitude of *V*_GS_ is smaller than that of the threshold voltage. In the weak inversion mode, the subthreshold leakage current is principally as a result of carriers' diffusion [58,59]. The off-state current of the transistor (*I*_OFF_) is the drain current when *V*_GS_ = 0. The off-state current is affected by some parameters such as channel length, channel width, depletion width of the channel, gate oxide thickness, threshold voltage, channel-source doping profiles, drain-source junction depths, supply voltage, and junction temperature [59].

**Figure 9:**
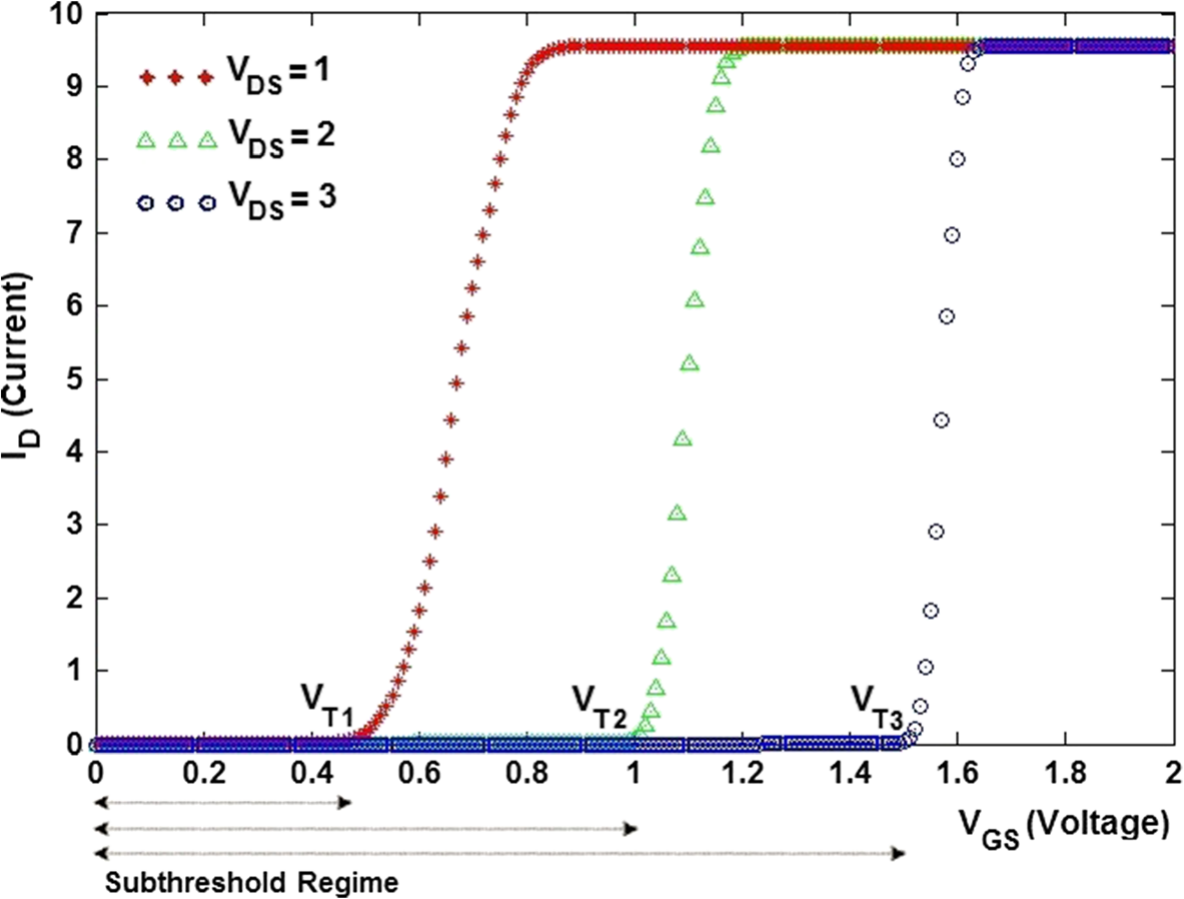
**Subthreshold regime of TGN SB FET at different values of *V***_**DS **_**(V) for *L *= 25 nm.**

Short-channel effects are defined as the results of scaling the channel length on the subthreshold leakage current and threshold voltage. The threshold voltage is decreased by reducing the channel length and drain-source voltage [58-61]. In the subthreshold region, the gate voltage is approximately linear [58,59]. It has been studied that the decrease of channel length and drain-source voltage results in shifting the characteristics to the left, and it is obvious that as the channel length gets less than 10 nm, the subthreshold current increases dramatically [62]. Based on the International Technology Roadmap for Semiconductors (ITRS) near-term guideline for low-standby-power technology, the value of the threshold voltage is close to 0.3 V [59]. Figure 9 illustrates the subthreshold regime of TGN SB FET at different values of drain-source voltage. As shown in this figure, for lower values of drain-source voltage, the threshold voltage is decreased and meets the guidelines of ITRS.

**Figure 10:**
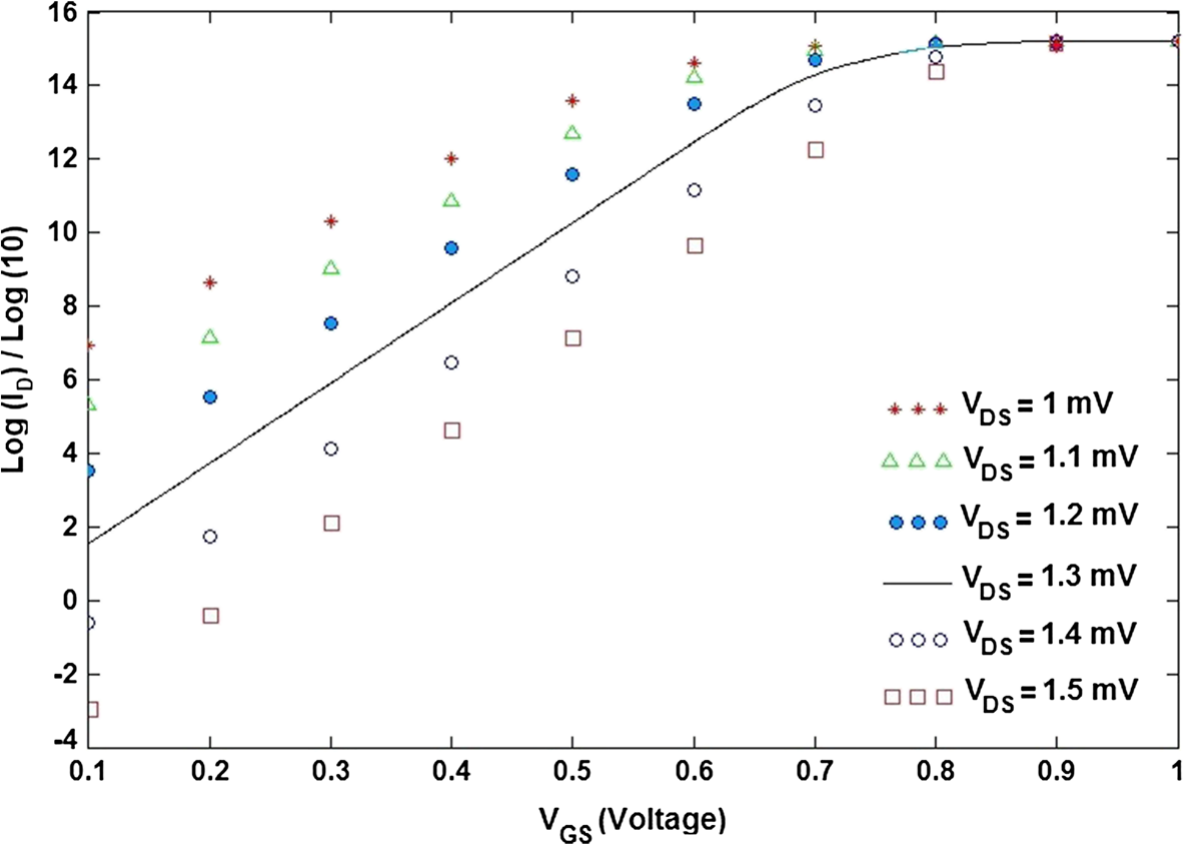
***I***_**D **_**(μA)-*V***_**GS **_**(V) characteristic of TGN SB FET at different values of *V***_**DS**_**.**

The subthreshold slope, *S* (mV/decade), is evaluated by selecting two points in the subthreshold region of an *I*_D_-*V*_GS_ graph as the subthreshold leakage current is adjusted by a factor of 10. It has been noted that self-consistent electrostatics and the gate bias-dependent electronic structure have an essential role in determining the intrinsic limits of the subthreshold slope in a TGN SB FET, which stays well over the Boltzmann limit of the ideal value of 60 mV/decade or less than 85mV/decade [58,63].The subthreshold slope, as one of the key issues of deep-submicrometer devices, is defined as [59]

(15) $$\mathrm{Subthreshold}\;\mathrm{slope}=\frac{V_{t}-V_{\mathrm{off}}}{\log\;I_{\mathrm{vt}}-\log\;I_{\mathrm{off}}}\text{,}$$

where *V*_t_ is the threshold voltage, *V*_off_ is the off voltage of the device, *I*_vt_ is the drain current at threshold, and *I*_off_ is the current at which the device is off. In other words, the subthreshold slope delineates the inverse slope of the log (*I*_D_) versus *V*_GS_ plotted graph as illustrated in Figure 10.

Average subthreshold swing is a fundamental parameter that influences the performance of the device as a switch. According to Figure 10, the subthreshold slope for (*l* = 100 nm) is obtained as shown in Table 1.

**Table 1:** **Subthreshold slope of TGN SB FET at different values of *V***_**DS**_

***V***_**DS **_**(mV)**	**1**	**1.1**	**1.2**	**1.3**	**1.4**	**1.5**
Subthreshold slope (mV/decade)	59.5238	54.1419	49.6032	45.8085	42.5134	39.2542

Based on data from [64], for the effective channel lengths down to 100 nm, the calculated and simulated subthreshold slope values are near to the classical value of approximately 60 mV/decade. The subthreshold slope can be enhanced by decreasing the value of the buried oxide capacitance *C*_BOX_ or by increasing the value of the gate oxide capacitance *C*_GOX_[64]. Based on the simulated results, it can be concluded that when the channel material is replaced by TGN, the subthreshold swing improves further.

The comparison study between the presented model with data from [62,64] showed that due to the quantum confinement effect [39,43], the value of the subthreshold slope in the case of TGN SB FET is less than those of DG metal oxide semiconductor and vertical silicon-on-nothing FETs [62,64] for some values of drain-source voltage. A nanoelectronic device characterized by a steep subthreshold slope displays a faster transient between on-off states. A small value of *S* denotes a small change in the input bias which can modulate the output current and thus leads to less power consumption. In other words, a transistor can be used as a high-speed switch when the value of *S* is small. As a result, the proposed model can be applied as a useful tool to optimize the TGN SB FET-based device performance. It showed that the shortening of the top gate may lead to a considerable modification of the TGN SB FET current–voltage properties. In fact, it also paves a path for future design of the TGN SB devices.

## Conclusions

TGN with different stacking arrangements is used as metal and semiconductor contacts in a Schottky transistor junction. The ABA-stacked TGN in the presence of an external electric field is also considered. Based on this configuration, an analytical model of junction current–voltage characteristic of TGN SB FET is presented. The dependence of the drain current versus the drain-source voltage of TGN SB FET as well as the back-gate and top-gate voltages for different values of gate-source voltage and geometric parameters such as channel length are calculated. In particular, we conclude that by increasing the applied voltage and also channel length, the drain current increases, which showed better performance in comparison with the typical behavior of other kinds of transistors. Finally, a comparative study of the presented model with MOSFET with a SiO_2_ gate insulator, a TGN MOSFET with an ionic liquid gate, and a TGN MOSFET with a ZrO_2_ wrap-around gate was presented. The proposed model is also characterized by a steep subthreshold slope, which clearly gives an illustration of the fact that the TGN SB FET shows a better performance in terms of transient between off-on states. The obtained results showed that due to the superior electrical properties of TGN such as high mobility, quantum transport, 1D behaviors, and easy fabrication, the suggested model can give better performance as a high-speed switch with a low value of subthreshold slope.

## Competing interests

The authors declare that they have no competing interests.

## Authors’ contributions

MR wrote the manuscript, contributed to the design of the study, performed all the data analysis, and participated in the MATLAB simulation of the proposed device. Prof. RI and Dr. MTA participated in the conception of the project, improved the manuscript, and coordinated between all the participants. HK, MS, and EA organized the final version of the cover letter. All authors read and approved the final manuscript.
